# Effect of poor glycaemic control on plasma levels and activity of protein C, protein S, and antithrombin III in type 2 diabetes mellitus

**DOI:** 10.1371/journal.pone.0223171

**Published:** 2019-09-27

**Authors:** Otchere Addai-Mensah, Max Efui Annani-Akollor, Frederick Obeng Nsafoah, Linda Ahenkorah Fondjo, Eddie-Williams Owiredu, Kwabena Owusu Danquah, Richard Vikpebah Duneeh, Francis Agyei Amponsah

**Affiliations:** 1 Department of Medical Diagnostics, Faculty of Allied Health Sciences, College of Health Sciences, Kwame Nkrumah University of Science and Technology, Kumasi, Ghana; 2 Department of Molecular Medicine, School of Medical Sciences, College of Health Sciences, Kwame Nkrumah University of Science and Technology, Kumasi, Ghana; 3 Department of Haematology, Komfo Anokye Teaching Hospital, Kumasi, Ghana; 4 St. John of God Hospital, Duayaw Nkwanta, Sunyani, Ghana; Medical University Innsbruck, AUSTRIA

## Abstract

**Background:**

Type 2 diabetes mellitus (T2DM) patients are predisposed to several diabetes-related complications. Dysregulation of the haemostatic mechanisms have been implicated. There are however no current studies assessing the levels and activity of protein C (PC), protein S (PS), and antithrombin III (AT III), which are essential in haemostatic regulation, in a single cohort of T2DM patients. This study evaluated the effect of poorly-managed T2DM on the levels and activity of PC, PS, and AT III.

**Methods:**

This cross-sectional study was conducted at the Diabetes Clinic, Cocoa Clinic in Kumasi, Ghana. A total of 242 T2DM patients, comprising 152 patients with poorly-managed diabetes and 90 well-managed diabetes patients, were recruited for the study. Fasting blood glucose, liver function tests and lipid profile were performed for each respondent. Glycated haemoglobin (HbA1c) was estimated by turbidimetric inhibition immunoassay. The levels and activity of PC, PS and AT III were measured by solid phase sandwich ELISA method.

**Results:**

There was a negative correlation between HbA1c and the levels and activity of PC, PS and AT III. The levels and activity of PC [(5.78 vs 4.64 μg/ml, p<0.0001) and (42.22 vs 36.21 U/ml, p = 0.01) respectively], PS [(22.55 vs 20.29 μg/ml, p = 0.010) and (235.94 vs 211.67 U/ml, p<0.0001) respectively] and AT III [(16.28 vs 14.41μg/ml, p<0.0001) and (176.01 vs 160.09 U/ml, p = 0.03) respectively] were significantly increased in patients with well-managed T2DM compared to the poorly-managed diabetes patients. Likewise, the levels and activity of PC, PS, and AT III was higher among T2DM patients using statins than patients who were statin-naïve. Among patients with well-managed T2DM, those who were on statins had significantly higher levels and activities of PC, PS, and AT III compared to well-managed T2DM patients not on statins. However, there no statistically significant differences between the level and activity of PC, PS, and AT III among poorly-managed T2DM patients with respect to statin status.

**Conclusion:**

Poorly-managed type 2 diabetes mellitus is associated with reduced levels and activity of PC, PS and AT III compared to well-managed T2DM. Though use of statins may improve the levels and activity of the PC, PS and AT III in T2DM, their effect is limited in the presence of poorly-controlled T2DM. Proper management of diabetes is essential to reduce the likelihood of thrombotic events among T2DM patients.

## Introduction

Type 2 Diabetes Mellitus (T2DM), a metabolic disease characterized by chronic hyperglycemia, altered insulin secretion, and impaired glucose tolerance, occurs as a result of insulin resistance and the inability of the pancreas to increase insulin production in the event of hyperglycaemia [1]. Its pervasive nature in recent decades has made it an epidemic, affecting over 500 million people globally [2] with over 6% of overweight urban Ghanaian adults affected [3, 1].

Micro- and macro-vascular complications are common [4, 5], with 80% of diabetic patients expiring from thrombotic events, a large majority (75–80%) of these deaths resulting from cardiovascular diseases [6, 7]. Additionally, a substantial number of these deaths have been linked to dysregulation of the haemostatic mechanisms in diabetes [8].

Natural anticoagulants such as Protein C (PC), Protein S (PS) and Antithrombin III (AT III) play pivotal roles in the regulation of the haemostatic pathway as they limit excess fibrin formation at the site of endothelial injury, thus abating the probability of plaque formation and its attendant thrombosis.

Evidence suggests that non-enzymatic glycation of the proteins may result in structural modifications which may culminate in protein dysfunction [9–11]. Consequently, glycosylation of the natural anticoagulants may result in deficiencies in their activity and levels, predisposing patients with persistent hyperglycaemia such as poorly-managed diabetics to an increased risk of thrombotic events [12]. Some studies have evaluated the levels of some coagulation parameters in T2DM and have reported reduced plasma levels of anti-thrombotic markers [8, 13, 14] and elevated levels of pro-thrombotic markers [15–18] with Erem *et al*. reporting an inverse association between glycated haemoglobin (HbA1c) and plasma protein S levels [18]. It is also known that hyperglycaemia induces antithrombin transition into a low heparin affinity form with a decrease in its anticoagulant activity [19].

Despite the knowledge of hyperglycaemia as one of the hallmarks of diabetes and its attendant effect on protein functionality, and the significance of hypercoagulability as a potential risk factor for diabetes-related complications, no study has assessed the effect of poor blood glucose management on natural anticoagulants in plasma. There are also no current studies assessing the levels and activity of PC, PS and AT III in a single cohort of T2DM patients. This study was thus conducted to evaluate the effect of poorly-managed T2DM on the levels and activity of PC, PS and AT III.

## Materials and methods

### Study design and setting

This was a hospital-based cross-sectional study conducted between May 2018 to January 2019 at the Diabetic Clinic of the Cocoa Clinic in Kumasi in the Ashanti region, Ghana. Kumasi is the second major city in Ghana [20] and has a projected population of 4,780,380, accounting for 19.4% of Ghana’s total population [21]. The estimated prevalence of T2DM in Ghana is 3.3–6%, the prevalence increasing with age and being higher in urban compared to the rural areas [22, 23]. In Kumasi, the prevalence of T2DM is 6% [24].

### Study population

The sample size for the study was calculated using Fischer’s sampling formula (*N* = *Z*^2^*PQ*/*d*^2^), where *Z* is the critical value of the normal distribution (1.96 at 95% CI); *P* is the estimated prevalence of T2DM in Kumasi (6%); *Q* = 100−*P*; and *d* is the absolute precision or sampling error tolerated = 5%. From the above equation, a total of 242 T2DM patients, comprising 152 patients with poorly-managed diabetes and 90 well-managed T2DM patients, were recruited for the study.

### Inclusion and exclusion criteria

Ghanaian adults between the ages of 30 and 70 years old with T2DM for at least one year were included in this study. Patients with a history of acute ischemic heart disease, cerebrovascular and peripheral vascular disease, and liver or kidney function impairment were excluded from the study. Participants with a history or family history of coagulation disorders were also excluded from the study. Patients with records of being on insulin injections were also excluded to limit the likelihood of recruiting type 1 diabetes patients.

### Questionnaire administration and clinical data extraction

A validated questionnaire was used to obtain socio-demographic data from the participants. Additional clinical data relevant to the study was extracted from the hospital’s archives.

### Anthropometric and blood pressure measurement

Weight was measured in the upright position to the nearest 0.1 kg using a calibrated balance beam scale. Height was measured (Subjects stood erect, barefoot, with feet together, looking forward) to the nearest 0.1 m using a measuring tape. Body Mass Index (BMI) was calculated as the observed weight in kilograms divided by the square of the height (in meters) (kg/m^2^) [25].

The blood pressure was measured with an automated blood pressure apparatus (Omron MX3-Omron Matsusaka Co., Ltd. Japan) from the right arm after the subject had been made to sit for at least five minutes. The average of the two readings taken five minutes apart was recorded. Hypertension was defined as a systolic blood pressure ≥ 140 mm Hg or diastolic blood pressure ≥ 90 mm Hg or history of previously known disease [26].

### Blood sample collection and processing

#### Blood sample collection

Six milliliters (6 ml) of venous blood was obtained under aseptic conditions with the participant seated. Two (2) milliliters of the blood was dispensed into a sodium fluoride tube for estimation of plasma glucose; another 2 ml was dispensed into a K_3_ EDTA tube for immediate estimation of HbA1c by turbidimetric inhibition immunoassay and the remaining 2 ml of blood dispensed into gel separator tubes. The remainder of the whole blood in the EDTA tubes and that in the gel separator tubes were centrifuged at 3000 rpm for 10 minutes within an hour of blood collection to obtain plasma and serum respectively. The aliquots were stored at -80 °C until analysis. The serum was used for lipid profile and liver function tests while the plasma was used for the measurement of the levels and activity of PC, PS, and AT III.

#### Biochemical measurements

The levels and activity of PC, PS and AT III were estimated using the solid phase sandwich ELISA. Briefly, 50 μL of standards, and 10 μL of samples and controls were pipetted into appropriate microtitre wells followed by 100 μL of enzyme conjugate reagent, mixed thoroughly, and covered with an adhesive strip. The microplate was incubated at 37°C for 60 minutes. The incubation mixture was then aspirated from the microplate wells followed by five washes with wash solution (400 μL). Residual water droplets were removed by striking the wells onto absorbent paper. A 100 μL of tetramethylbenzidine (TMB) solution (prepared from 50 μL of chromogen A and 50 μL of chromogen B according to the manufacturer’s instructions) was then pipetted into each well, mixed gently and incubated at 37°C for 15 minutes. The reaction was stopped by adding 50 μL of Stop Solution to each well and gently mixed for 30 seconds. The absorbance of the final colored product was measured spectrophotometrically at 450 nm using Thermo Electron Multiskan EX plate reader (Shanghai, China). The mean absorbance value (OD_450_) for each set of reference standards, controls and samples were calculated. The calculated mean OD_450_ obtained for each reference standard were used to construct a standard curve and the concentrations of samples and controls determined from the standard curve.

Estimation of lipid profile, liver function tests and glycated haemoglobin (HbA1c) levels were performed using the Cobas Integra automated Chemistry analyzer (Roche Cobas Integra 400 Plus, Roche Diagnostics, USA). Lipid profile and liver function was measured spectrophotometrically.

Whole blood was used for HbA1c estimation by turbidimetric inhibition immunoassay as previously described [27, 28]. Briefly, well-mixed EDTA-anticoagulated whole blood was put into sample tubes. The tubes were immediately placed on a rack. Red blood cells are then haemolysed by low osmotic pressure and the free haemoglobin subsequently degraded by pepsin to ensure availability of the N-terminal of the beta chain (β-N-terminal) of haemoglobin. Latex particle-bound monoclonal antibodies bind to the β-N-terminal of HbA1c while the remaining free antibodies are agglutinated using synthetic polymers with multiple copies of the β-N-terminal structure of HbA1c. The change in turbidity is measured at 552 nm and the final HbA1c value expressed as a percentage using the formula: HbA1c (%) = (HbA1c/Hb) × 87.6 + 2.27. The test was standardized with an intra-assay % CVs of 0.9%-1.5% and inter-assay %CVs of 1.1%-1.6%). Daily calibration and maintenance of the analyzer was performed according to the manufacturer’s instructions as previously described [29]. Quality control (QC) was assessed using quality control materials provided by the manufacturer [negative and positive controls (high and low HbA1c)] and calibration was performed using manufacturer-supplied calibrator (Cfas HbA1c). Patients with HbA1c ≥ 8.0% were considered to have poorly-managed T2DM while patients with HbA1c < 8% were considered to have good glycaemic control/ well-managed T2DM [30–32].

### Data analysis

The data obtained was entered and cleaned in Microsoft Excel 2016 prior to analysis. Categorical data were presented as frequency (percentages) and Chi square and Fisher’s exact test statistic were used to test for association where applicable. Test for normality was performed using Shapiro-Wilk test. Continuous variables were presented as mean ± standard deviation for parametric variables and median (inter-quartile range) for non-parametric variables. Independent t-test and Mann-Whitney U test was used to test for significance of differences between parametric and non-parametric variables respectively. Non-parametric one-way analysis of covariance (ANCOVA) was used to assess significance of differences in the levels and activity of plasma anticoagulants by glycaemic and statin status after controlling for BMI. Spearman’s correlation was employed to assess associations between anthropometric, haemodynamic and biochemical parameters with the coagulation parameters. A *p* value < 0.05 was considered statistically significant. All statistical analyses were performed using Stata/MP version 13.0 and GraphPad Prism 7 version 7.04.

## Results

Table 1 shows the baseline characteristics of the entire study population. The average age and duration of T2DM of the study population was 58.74 years and 4.87 years respectively. The proportion of male and female participants were 68.6% and 31.4% respectively. The mean HbA1c was 8.87% and the median levels and activity of PC, PS, and AT III were 5.04 μg/ml and 37.33 U/ml, 20.29 μg/ml and 212.58 U/ml, 14.41 μg/ml and 160.09 U/ml respectively (Table 1).

**Table 1:** Baseline characteristics of the study participants.

Variables	Frequency (%)
**Demographic characteristics**	
Age (years)	58.74 ± 8.51
Sex	
Female	76 (31.4)
Male	166 (68.6)
**Clinical characteristics**	
Frequency of Physician appointment	
Bi-monthly	126 (52.1)
Monthly	116 (47.9)
Treatments	
Metformin	218 (90.1)
Sulphonylureas	146 (60.3)
Statins	114 (47.1)
Duration of T2DM (years)	4.87 ± 4.28
**Anthropometry and haemodynamic characteristics**	
Height (m)	1.65 ± 0.08
Weight (kg)	72.72 ± 13.24
BMI (kg/m2)	26.61 ± 4.87
SBP (mmHg)	135.75 ± 15.54
DBP (mmHg)	80.89 ± 12.91
**Biochemical profile**	
FBG (mmol/L)	9.27 ±4.02
HbA1c (%)	8.87 ± 1.88
TC (mmol/l)	5.24 ± 1.58
TG (mmol/L)	1.55 ± 0.61
HDL-C (mmol/L)	1.27 ± 0.22
LDL-C (mmol/L)	3.99 ± 1.11
ALT (IU/L)	10.00 ± 8.13
AST (IU/L)	10.01 ± 7.30
ALP (IU/L)	123.05 ± 56.62
Albumin (g/L)	41.85 ± 18.30
TP (g/L)	63.54 ± 4.60
**Haematological profile**	
Protein C level (μg/ml)	5.04 (4.34–5.94)
Protein C activity (U/ml)	37.33 (33.39–42.22)
Protein S level (μg/ml)	20.29 (16.99–23.31)
Protein S activity (U/ml)	212.58 (197.61–237.58)
Antithrombin III level (μg/ml)	14.41 (11.97–17.50)
Antithrombin III activity (U/ml)	160.09 (140.90–181.37)

BMI; Body mass index, SBP; Systolic blood pressure, DBP; Diastolic blood pressure, FBG; Fasting blood glucose, HbA1c; Glycated haemoglobin, TC; Total cholesterol, TG; Triglyceride, HDL-C; High density lipoprotein cholesterol, LDL-C; Low density lipoprotein cholesterol, ALT; Alanine transaminase; AST; Aspartate transaminase, ALP; Alkaline phosphatase, TP; Total protein

The baseline characteristics of the study population stratified by T2DM management status is shown in Table 2. A total of 152 patients with poorly-managed T2DM and 90 well-managed T2DM patients were included in the study. While patients with poorly-managed T2DM presented with significantly elevated fasting blood glucose (10.06 mmol/l vs 6.78 mmol/l, p = 0.003) and HbA1c levels (10.01% vs 6.94%, p<0.0001), subjects with well-managed T2DM had higher BMI and diastolic blood pressure compared to the poorly-managed group although their mean diastolic pressures were within the normal range. There were no significant differences between well-managed and poorly managed T2DM with respect to the lipid profile and liver function tests (Table 2).

**Table 2:** Baseline characteristics of the study population stratified by T2DM management status.

Variables	Well-managed (n = 90)	Poorly-managed (n = 152)	p-value
**Demographic characteristics**			
Age (years)	58.78 ± 8.72	58.72 ± 8.42	0.962‡
Sex			0.668†
Female	30 (39.5)	46 (60.5)	
Male	60 (36.1)	106 (63.9)	
**Clinical characteristics**			
Frequency of Physician appointment			0.185†
Bi-monthly	52 (41.3)	74 (58.7)	
Monthly	38 (32.8)	78 (67.2)	
Treatments			
Metformin	88 (40.4)	130 (59.6)	**0.001**†
Sulphonylureas	32 (21.9)	114 (78.1)	**<0.0001**†
Statins	20 (17.5)	94 (82.5)	**<0.0001**†
Duration of T2DM (years)	4.76 ± 4.35	4.98 ± 3.21	0.653‡
**Anthropometry and haemodynamic characteristics**			
Height (m)	1.68 ± 0.07	1.64 ± 0.08	**0.0010**‡
Weight (kg)	79.45 ± 11.98	68.74 ± 12.34	**<0.0001**‡
BMI (kg/m2)	28.54 ± 5.62	25.47 ± 3.96	**<0.0001**‡
SBP (mmHg)	138.22 ± 14.94	134.29 ± 15.76	0.057‡
DBP (mmHg)	83.78 ± 8.87	79.18 ± 14.55	**0.007**‡
**Biochemical profile**			
FBG (mmol/l)	6.78 ±0.68	10.06 ±1.54	**0.003**
HbA1c (%)	6.94 ±0.67	10.01 ± 1.36	**<0.0001**‡
TC (mmol/l)	5.56 ±1.21	5.60 ±1.27	0.889‡
TG (mmol/l)	1.53 ±0.46	1.56 ±0.74	0.824‡
HDL-C (mmol/l)	1.29 ±0.23	1.25 ±0.20	0.407‡
LDL-C (mmol/l)	3.96 ±1.08	4.02 ±1.15	0.827‡
ALT (IU/L)	9.49 ±7.47	10.54 ±8.85	0.594‡
AST (IU/L)	10.04 ±8.17	10.00 ± 6.38	0.976‡
ALP (IU/L)	120.68 ±50.96	125.56 ±62.75	0.721‡
Albumin (g/L)	39.59 ±3.03	44.24 ±26.06	0.292‡
TP (g/L)	64.13 ±3.13	62.92 ±5.74	0.276‡

Well-managed T2DM; HbA1c < 8%, Poorly-managed T2DM; HbA1c ≥ 8.0%, BMI; Body mass index, SBP; Systolic blood pressure, DBP; Diastolic blood pressure, FBG; Fasting blood glucose, HbA1c; Glycated haemoglobin, TC; Total cholesterol, TG; Triglyceride, HDL-C; High density lipoprotein cholesterol, LDL-C; Low density lipoprotein cholesterol, ALT; Alanine transaminase; AST; Aspartate transaminase, ALP; Alkaline phosphatase, TP; Total protein, p-value <0.05 was considered statistically significant (p-values of significant variables in bold print)

^‡^; Independent t-test

^†^; Fisher exact test

Table 3 shows the correlation analysis between the levels and activity of protein C, protein S and antithrombin III with biochemical, haemodynamic and anthropometric measurements. There were negligible correlations between the levels and activity of PC, PS and AT III with biochemical, haemodynamic and anthropometric measurements. A weak negative correlation was found between HbA1c and PS activity and AT III levels (Table 3).

**Table 3:** Correlation analysis between the levels and activity of PC, PS, AT III and the biochemical, haemodynamic and anthropometric measurements.

Variables	PC level	PC activity	PS level	PS activity	AT III level	AT III activity
FBG (mmol/l)	0.100	0.035	0.083	0.054	0.088	0.044
HbA1c (%)	-0.201	-0.182	-0.232	**-0.327**	**-0.325**	-0.165
TC (mmol/l)	0.116	-0.045	0.221	0.037	-0.068	-0.162
TG (mmol/l)	0.074	0.001	**0.250**	0.075	0.118	0.065
HDL-C (mmol/l)	-0.011	-0.073	0.085	-0.099	0.204	**0.297**
LDL-C (mmol/l)	-0.061	-0.095	-0.028	-0.076	0.071	-0.007
Albumin (g/L)	-0.006	0.028	-0.027	0.064	0.213	-0.134
TP (g/L)	-0.154	-0.093	0.080	-0.078	0.048	0.103
ALP (IU/L)	0.103	0.193	0.004	0.065	0.045	-0.07
AST (IU/L)	0.098	0.066	0.138	0.066	0.061	0.142
ALT (IU/L)	0.123	0.089	0.118	0.062	-0.081	0.057
BMI (kg/m^2^)	0.16	0.157	-0.291	0.222	-0.108	**-0.274**
SBP (mmHg)	0.167	-0.007	0.197	0.112	0.292	0.187
DBP (mmHg)	0.206	0.265	0.233	0.212	0.208	0.220

Spearman’s correlation was performed to evaluate the association between the natural anticoagulants and anthropometric, haemodynamic and biochemical parameters after controlling for age and sex. BMI; Body mass index, SBP; Systolic blood pressure, DBP; Diastolic blood pressure, FBG; Fasting blood glucose, HbA1c; Glycated haemoglobin, TC; Total cholesterol, TG; Triglyceride, HDL-C; High density lipoprotein cholesterol, LDL-C; Low density lipoprotein cholesterol, ALT; Alanine transaminase; AST; Aspartate transaminase, ALP; Alkaline phosphatase, TP; Total protein. Correlations with significant *p*-values in bold print

**Fig 1:**
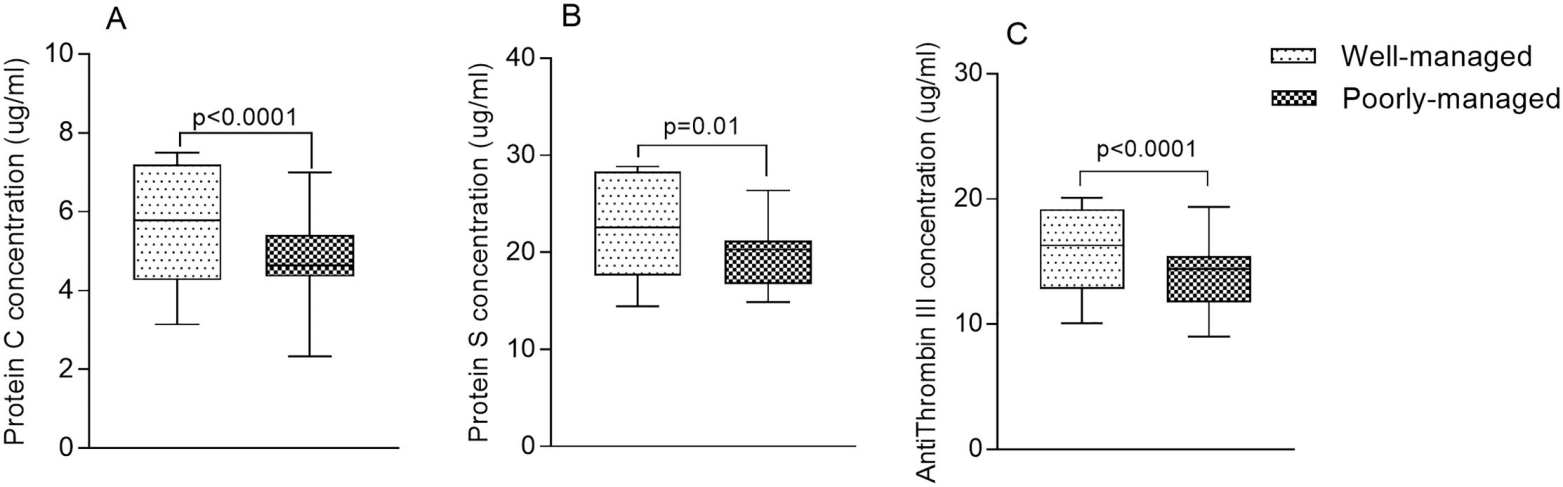
The levels of protein C, protein S and antithrombin III stratified by T2DM management status.

The levels of PC [5.78 μg/ml (4.26–7.19) vs 4.64 μg/ml (4.35–5.41) respectively, p<0.0001] (Fig 1A), PS [22.55 μg/ml (17.61–28.31) vs 20.29 μg/ml (16.72–21.23) respectively, p = 0.010] (Fig 1B) and AT III [16.28 μg/ml (12.82–19.18) vs 14.41μg/ml (11.75–15.44) respectively, p<0.0001] (Fig 1C) were significantly higher in patients with well-managed T2DM compared to patients with poorly-managed T2DM (Fig 1).

**Fig 2:**
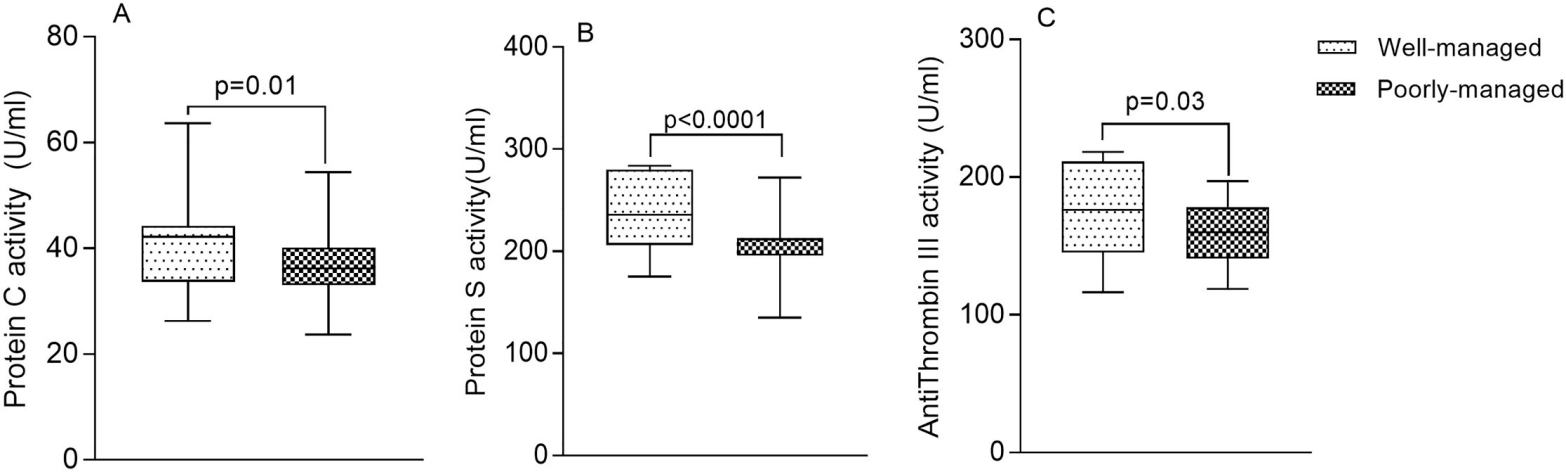
The activity of protein C, protein S and antithrombin III stratified by T2DM management status.

As shown in Fig 2, the activity of PC [42.22 U/ml (33.69–44.33) vs 36.21 U/ml (33.0–40.19) respectively, p = 0.01] (Fig 2A), PS [235.94 U/ml (206.11–279.71) vs 211.67 U/ml (195.68–213.03) respectively, p<0.0001] (Fig 2B) and AT III [176.01 U/ml (145.0–211.47) vs 160.09 U/ml (140.99–178.04) respectively, p = 0.03] (Fig 2C) were significantly elevated in patients with well-managed T2DM compared to patients with poorly-managed T2DM (Fig 2).

**Fig 3:**
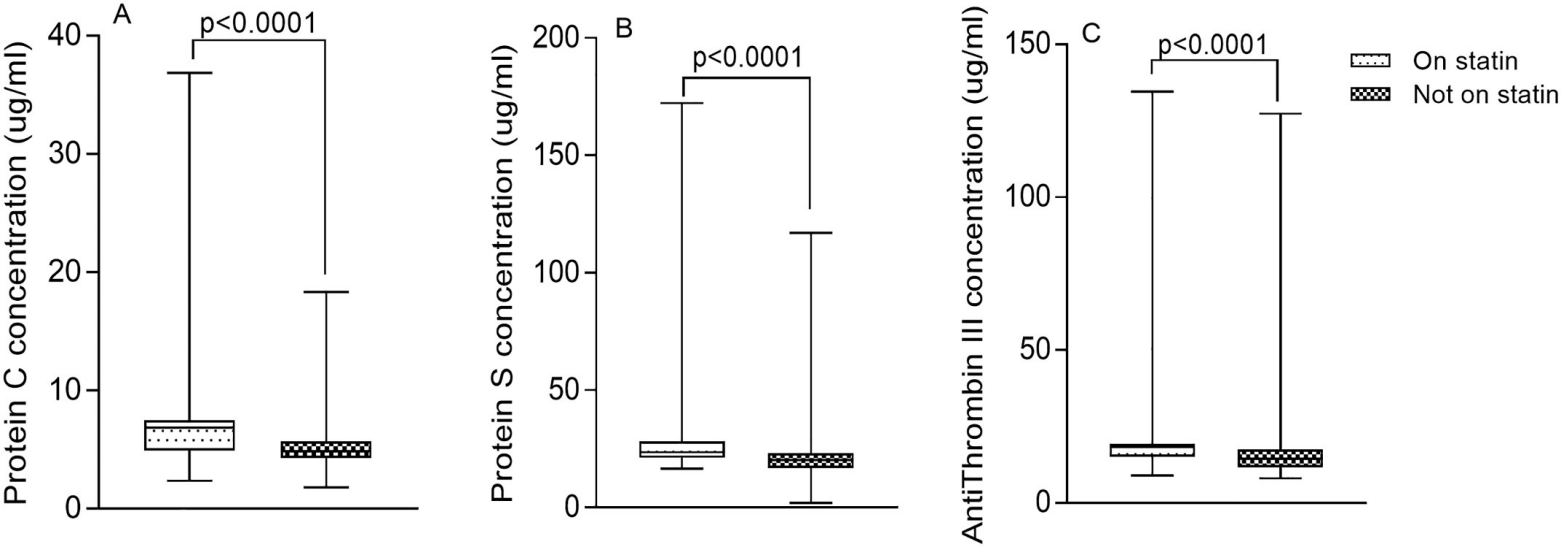
The levels of Protein C, Protein S and Antithrombin III stratified by statin use.

The levels of PC [6.85 μg/ml (4.92–7.49) vs 4.85 μg/ml (4.29–5.67) respectively, p<0.0001] (Fig 3A), PS [23.61 μg/ml (21.18–28.31) vs 20.29 μg/ml (16.96–23.14) respectively, p<0.0001] (Fig 3B) and AT III [18.26 μg/ml (15.07–19.32) vs 14.41 μg/ml (11.75–17.41) respectively, p<0.0001] (Fig 3C) were significantly increased in patients who were on statins compared to patients who were statin-naïve (Fig 3).

**Fig 4:**
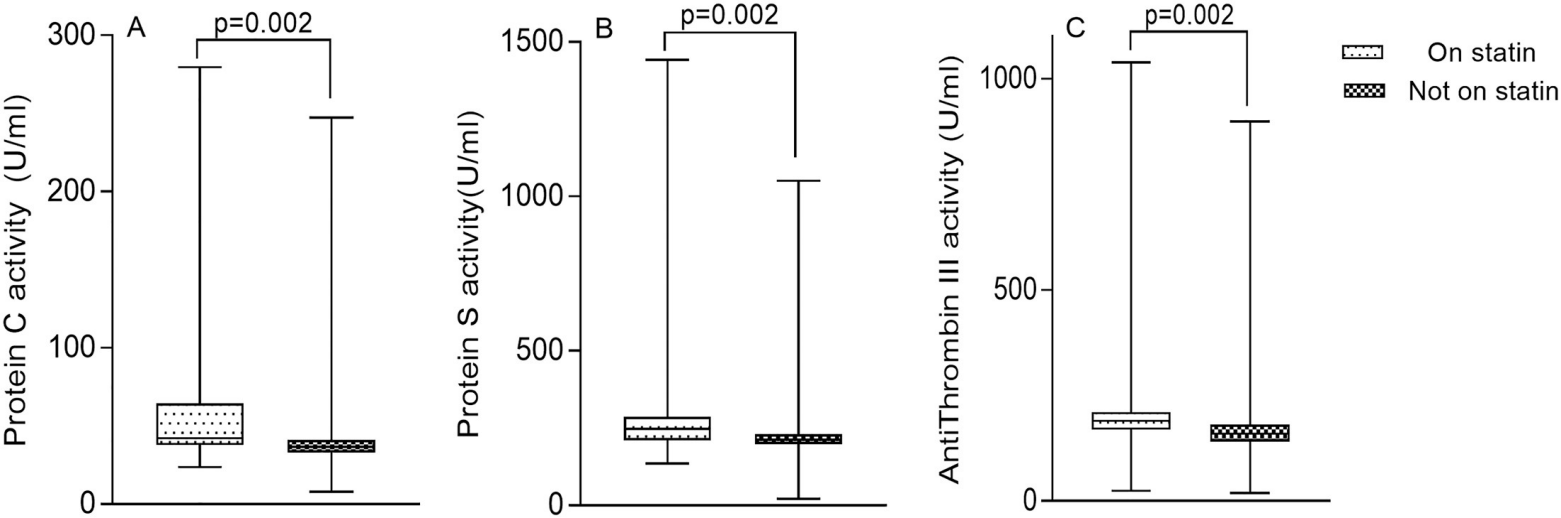
The activity of Protein C, Protein S and Antithrombin III stratified by statin use.

The activity of PC [42.22 U/ml (37.53–64.46) vs 36.73 U/ml (32.82–41.18) respectively, p = 0.002] (Fig 4A), PS [248.25 U/ml (210.65–286.79) vs 211.67 U/ml (197.55–230.21) respectively, p = 0.002] (Fig 4B) and AT III [190.05 U/ml (169.28–211.47) vs 160.09 U/ml (140.99–181.37) respectively, p = 0.002] (Fig 4C) were significantly increased in patients who were on statins compared to patients who were not on statins (Fig 4).

**Fig 5:**
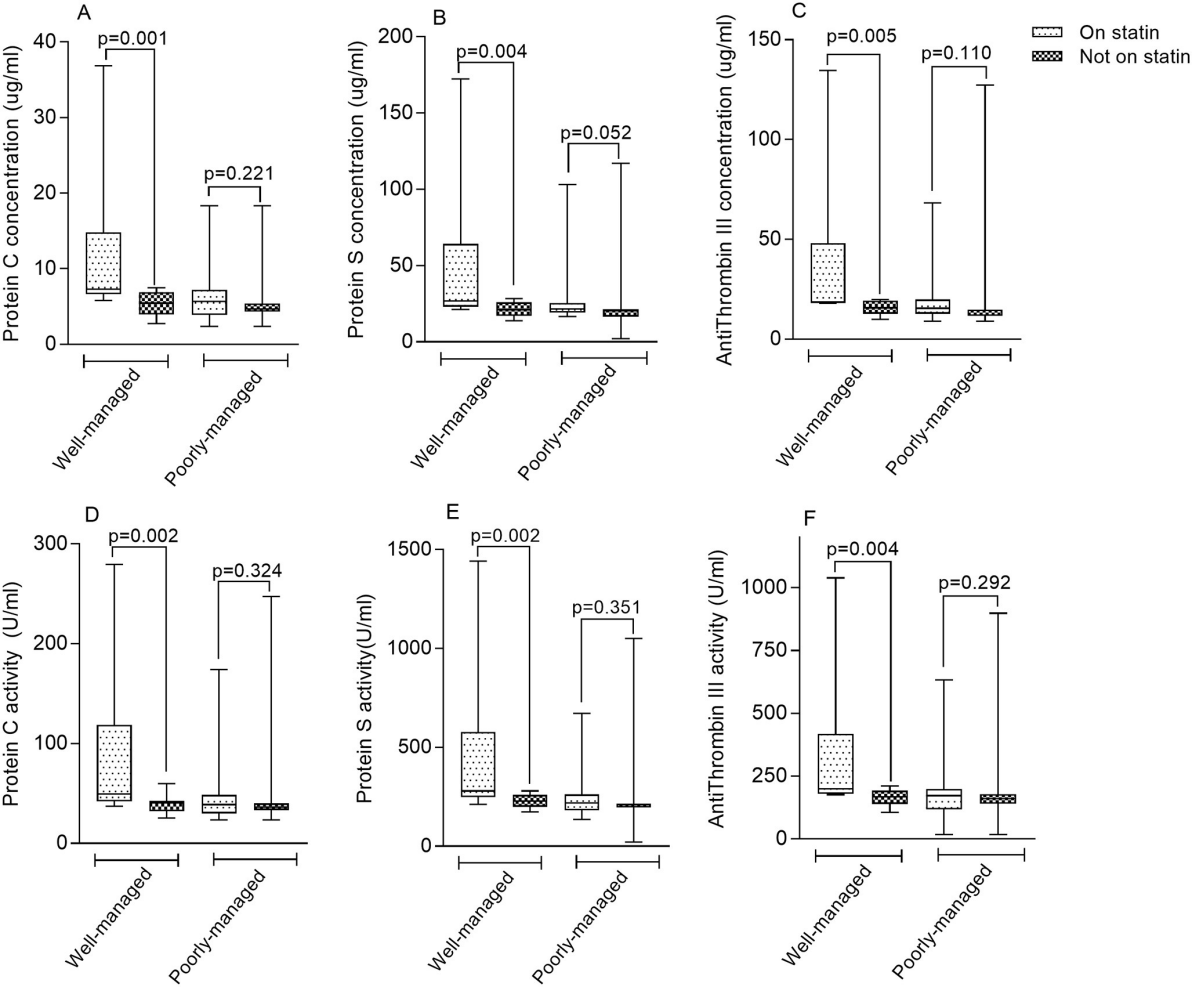
Levels and Activity of Protein C, Protein S and Antithrombin III among patient with well- and poorly-managed T2DM stratified by statin use.

Among patients with well-managed T2DM, those who were on statins had significantly higher levels of PC [7.30 μg/ml (6.67–14.83) vs 5.45 μg/ml (3.95–6.93) respectively, p = 0.001] (Fig 5A), PS [26.68 μg/ml (23.04–64.31) vs 21.03 μg/ml (16.99–25.93) respectively, p = 0.004] (Fig 5B), and AT III [18.75 μg/ml (18.11–48.02) vs 15.70 μg/ml (12.72–19.18) respectively, p = 0.005] (Fig 5C) compared to patients not on statins. Similarly, the activity of PC [49.25 U/ml (42.22–118.76) vs 40.64 U/ml (32.42–42.96) respectively, p = 0.002] (Fig 5D), PS [276.71 U/ml (248.52–577.36) vs 228.0 U/ml (198.97–259.30) respectively, p = 0.002] (Fig 5E) and AT III [200.84 U/ml (180.39–418.41) vs 168.13 U/ml (139.79–192.10) respectively, p = 0.004] (Fig 5F) were significantly increased among well-managed T2DM patients using statins compared to well-managed T2DM patients not on statins. There were however no statistically significant differences between the levels and activity of PC, PS and AT III among patients with poorly-managed T2DM with respect to statin status (Fig 5).

## Discussion

Patients with type 2 diabetes mellitus (T2DM) are predisposed to several T2DM-related complications [33]. An important type being microvascular complications such as diabetic retinopathy, neuropathy, and nephropathy. These complications have been linked with the imbalance between the haemostatic and thrombosis protecting systems in diabetic patients, resulting in a hypercoagulable state, and this is partly linked with hyperglycaemia [34]. Previous studies have reported significantly increased levels of pro-thrombotic factors in the plasma of T2DM patients compared to non-diabetic controls [15–17]. Other studies have also reported reduced levels of anti-thrombotic factors in T2DM. In a case-control study among T2DM patients in Turkey, Aslan *et al*. observed a significantly reduced level of plasma protein C (PC) in the cases than healthy controls [8]. Fattah *et al*. also reported reduced PC levels and activity in non-obese non-insulin dependent diabetes patients in comparison with non-obese healthy control [14]. A case-control study by Kim *et al*. also observed a significantly reduced PC levels in T2DM patients compared to healthy controls [13]. A study by Kath *et al*. reported no statistically significant difference in PC levels among the T2DM patients compared to a healthy control group. However, plasma levels of PS and AT III were remarkably lower among the cases than controls [35]. In another study, Erem *et al*. observed that the levels of glycated haemoglobin (HbA1c) correlated inversely with plasma protein S (PS) activity [18]. The evidence thus suggests that persistently high blood glucose levels may play a pivotal role in the haemostatic derangements in T2DM patients. However, despite the influence of blood glucose levels on natural anticoagulants which play a seminal role in the regulation of haemostasis, no current study has elucidated the effect of glycaemia management on the plasma levels and activity of these natural anticoagulants. The present study assessed the effect of glycaemia management on the plasma levels and activity of PC, PS, and antithrombin III (AT III) among type 2 diabetic patients.

In order to elucidate the influence of T2DM management on the level and activity of the natural anticoagulants, subjects were stratified into well-managed and poorly-managed diabetes based the level of HbA1c. Interestingly, well-managed T2DM patients had significantly higher levels and activity of PC, PS and AT III compared to those that were poorly-managed, suggesting that poor management of glycaemia may predispose T2DM patients to a hypercoagulable state compared to well-managed T2DM. This finding could be attributed to the possible influence of prolonged hyperglycaemia, evidenced by elevated HbA1c, on the levels and activity of PS, PC and AT III. Accumulating evidence suggest that non-enzymatic glycation of various protein may result in structural modifications which may culminate in protein dysfunction [9–11]. There are also reports that chronic hyperglycemia results in excess formation of non-enzymatic glycosylation products which have been attributed to development of long-term diabetic complications [36]. Consequently, glycosylation of the natural anticoagulant, in this case, PC, PS and AT III may have resulted in deficiencies in their activity and levels, thus predisposing patients with poorly-managed diabetics and persistent hyperglycaemia to the risk of thrombotic events [12]. Buttressing this finding is the negative correlation we observed between HbA1c and the levels and activity of the natural anticoagulants.

In addition to hyperglycaemia, arterial hypertension and hyperlipidaemia also commonly occur in patients with T2DM. Due to the close association of hyperglycaemia, arterial hypertension and hyperlipidaemia with the development of cardiovascular events, medications such as statins are commonly prescribed to T2DM patients. In a study by Kim *et al*., it was reported that T2DM patients receiving statins had elevated levels of PC and a relative amelioration of the hypercoagulable state [37]. To explore this association in the present study population, patients were re-stratified into those on statins and those who are statin-naïve.

We found that the levels and activity of PC, PS and AT III were significantly increased in T2DM patients who were on statins compared to patients who were statin-naïve; supporting the disposition that statins not only exert direct effects on lipids, but also influences coagulation, reducing the risk of thrombotic events among T2DM patients [38, 39]. This finding is also in harmony with a recent cross-sectional study by Aktas *et al*. among T2DM patients receiving therapy with statins and ACE inhibitors or angiotensin II receptor blockers in Turkey [40]. Furthermore, the elevated PC in this present study may be attributed to the influence of statins on the PC pathway. It has been demonstrated that statins increase the expression of plasma thrombomodulin in the endothelium and, thus, increase PC activation, which results in a potential in vivo antithrombotic effect [39]. The mechanism underpinning the influence of statins on PS and AT III is yet to be fully elucidated. However, proposed mechanisms include suppression of thrombin generation which has been reported to have inhibitory effect on PC, PS, and AT III [39].

It is evident that, well-controlled T2DM and the use of statins positively influence the levels and activity of the natural anticoagulants. However, the concomitant influence of both glycaemia management and use of statins is yet to be explored. Patients with well-managed T2DM were grouped into those that used statins and those who were statin-naïve. A similar stratification was performed for poorly-managed T2DM.

Interestingly, among the patients with well-managed T2DM, subjects on statins had significantly higher levels and activity of PC, PS, and AT III compared to patients not on statins. Conversely, however, we found no statistically significant differences between the levels and activity of PC, PS and AT III among patients with poorly-managed T2DM with respect to statins status. This finding suggests that, though statin use may play a significant role in improving the levels and activity of the PC, PS and AT III in T2DM, ultimately abating the risk of thrombotic events, their effect is limited in the presence of poorly-controlled T2DM.

We thus recommend proper glycaemia management as a way to minimize the propensity of thrombotic events among T2DM patients.

The major limitation of this study is its cross-sectional design as this precluded the establishment of causal relationships between glucose control and the levels and activity of PC, PS and AT III. Further studies to confirm this association is warranted. These studies may consider additional markers of hyperaggregation, vasoconstriction or inflammation which were not included in this study.

## Conclusion

Poorly-managed T2DM is associated with reduced levels and activity of protein C, protein S and antithrombin III compared to well-managed T2DM. Though use of statins may improve the levels and activity of the PC, PS and AT III in T2DM, their effect is limited in the presence of poorly-controlled T2DM. Proper management of T2DM is essential to reduce the likelihood of thrombotic events among T2DM patients.

## Ethics approval and consent to participate

Ethical approval for this study was obtained from the Committee on Human Research Publication and Ethics (CHRPE) of the School of Medical Sciences, Kwame Nkrumah University of Science and Technology. Written informed consent was obtained from all participants who opted to participate after the aims and objectives of the study had been explained to them. Participation was voluntary, and respondents were assured that the information obtained was strictly for research and academic purposes only and were guaranteed the liberty to opt out from the study at their own convenience.

## Supporting information

S1 Dataset Excel sheet of dataset on which the conclusions of this manuscript were made. (XLSX)
